# Health equity and migrants in the Greater Mekong Subregion

**DOI:** 10.1080/16549716.2017.1271594

**Published:** 2017-01-27

**Authors:** Celia McMichael, Judith Healy

**Affiliations:** ^a^ The University of Melbourne, School of Geography, Carlton, Australia; ^b^ Australian National University, School of Regulation and Global Governance (RegNet), Canberra, Australia

**Keywords:** Migrant, migration, universal health coverage, Greater Mekong Subregion, South-Eastern Asia, health equity

## Abstract

**Background**: Migrant health is receiving increasing international attention, reflecting recognition of the health inequities experienced among many migrant populations and the need for health systems to adapt to diverse migrant populations. In the Greater Mekong Subregion (GMS) there is increasing migration associated with uneven economic integration and growth, socio-economic vulnerabilities, and disparities between countries. There has been limited progress, however, in improving migrant access to health services in the Subregion. This paper examines the health needs, access barriers, and policy responses to cross-border migrants in five GMS countries.

**Methods**: A review of published literature and research was conducted on migrant health and health service access in Cambodia, Lao People’s Democratic Republic, Myanmar, Thailand, and Viet Nam, as well as analysis of current migration trends and universal health coverage (UHC) indicators in the Subregion. The review included different migrant types: i.e. migrant workers, irregular migrants, victims of trafficking, refugees and asylum seekers, and casual cross-border migrants.

**Results**: There is substantial diversity in the capacity of GMS health systems to address migrant populations. Thailand has sought to enhance migrant health coverage, including development of migrant health policies/programs, bilateral migrant worker agreements, and migrant health insurance schemes; Viet Nam provides health protection for emigrant workers. Overall, however, access to good quality health care remains weak for many citizens in GMS countries let alone migrants. Migrant workers – and irregular migrants in particular – face elevated health risks yet are not adequately covered and incur high out-of-pocket (OOP) payments for health services.

**Conclusions**: UHC implies equity: UHC is only achieved when *everyone* has the opportunity to access and use good-quality health care. Efforts to achieve UHC in the GMS require deliberate policy decisions to include migrants. The emergence of the UHC agenda, and the focus on migrant health among policy makers and partners, present an opportunity to tackle barriers to health service access, extend coverage, and strengthen partnerships in order to improve migrant health. This is an opportune time for GMS countries to develop migrant-inclusive health systems.

## Background

### Migrants and health equity in the Greater Mekong Subregion

Health inequities are common among disadvantaged population subgroups, including migrants, a group who commonly encounter many forms of disadvantage. With increasing population mobility and displacement in our rapidly globalising world, migrants warrant more attention if countries are to meet the Sustainable Development Goals (SDGs) by 2030, in particular Goal 10 which aims to ‘reduce inequality within and among countries’, and Goal 3 that aims to ‘ensure healthy lives and promote well-being for all at all ages’ [1]. Migrants, especially irregular migrants, experience considerable health inequity, defined as ‘unjust and avoidable differences in health that stem from some form of discrimination or lack of access to certain resources’ [2]. Improving health care is a key pathway towards health equity, as set out in SDG target 3.8 which aims to ‘achieve universal health coverage (UHC), including financial risk protection, access to quality essential health care services, and access to safe, effective, quality, and affordable essential medicines and vaccines for all’ [1]. Achieving UHC will require low- and middle-income countries, in particular, to strengthen their health care systems [3] and to pay more attention to the broader goal of health equity [4,5].

In 2010, the Asia-Pacific region had an estimated 27.5 million international migrants and an unknown number of irregular migrants [6]. Yet there has been limited progress in improving migrant health and access to health services, and documents surrounding SDGs and UHC seldom specifically mention migrants. The 2010 Global Consultation on Migrant Health directed attention to the need to develop migrant-sensitive health systems [7], however, and migrants are now receiving more attention under World Health Organization (WHO) regional frameworks for advancing SDGs and UHC in the Asian region [8,9].

Migrant health, however, remains a vexed issue in low- and middle-income countries that struggle to provide adequate health services for their citizens, let alone migrants who are often regarded as having a lesser claim. Cross-border migrants – particularly irregular migrants – are difficult to count, typically have multiple health needs, and are seldom embraced as a responsibility by either origin or destination countries in Asia.

**Figure 1.**
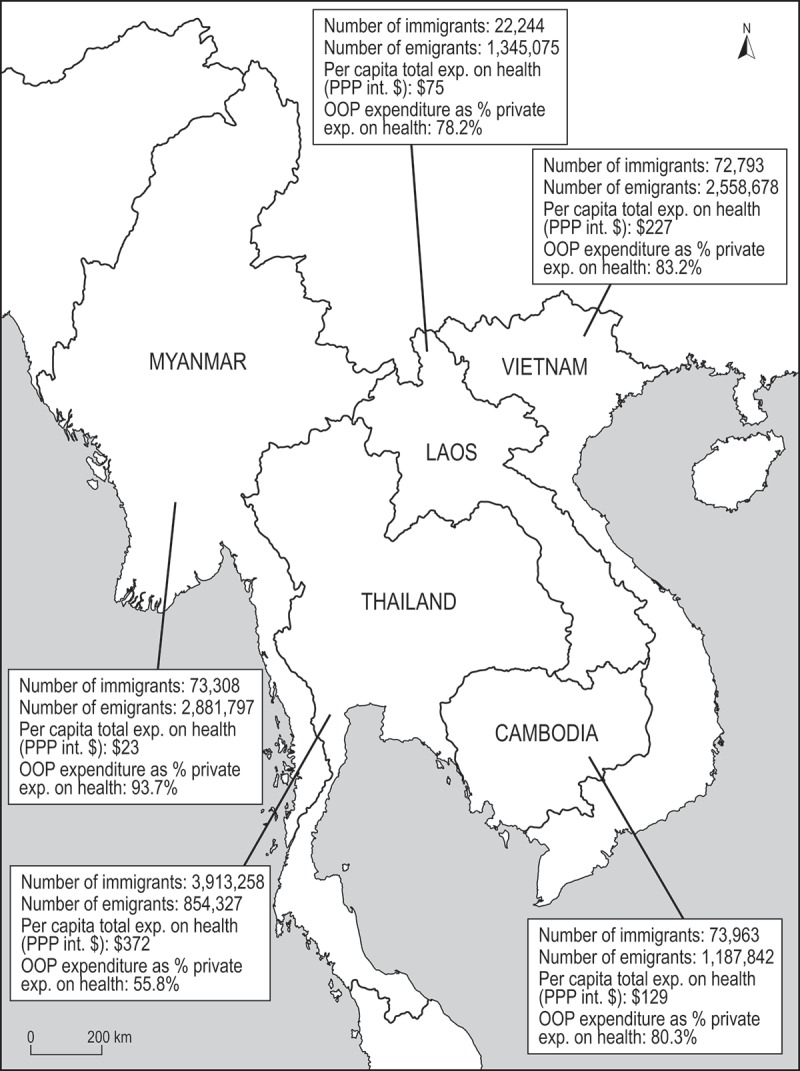
The Greater Mekong Subregion (GMS). Data sources: [10–18].

This paper examines the health needs, access barriers, and policy responses of relevance to cross-border migrants in the Greater Mekong Subregion (GMS) (see Figure 1). While the focus is on international migration in the GMS, key messages are relevant to other regions with migrant populations. The paper concentrates on the role of destination-country health systems, drawing on examples from Cambodia, Lao People’s Democratic Republic (Lao PDR), Myanmar, Thailand, and Viet Nam, while recognising that migrant health requires action from both origin countries and destination countries, as well as via bilateral and regional collaborations.

## Methods

The paper focuses on international migrants, including international labour migrants and irregular migrants; internal migrants (i.e. people displaced within national boundaries) are not included in the analysis. Five GMS countries are included for analysis: Cambodia, Lao PDR, Myanmar, Thailand, and Viet Nam. The analysis does not include Yunnan Province and Guangxi Zhuang Autonomous Region of Southern China (also regarded as part of the GMS). As this is an emerging area of policy focus and research, a scoping review approach was used. Reviewed literature included published articles, book chapters, reports and publications by international institutions, policy documents, and media articles. The search strategy included Google, Google Scholar, Web of Science, PubMed, and WHO websites. Key search terms were selected based on the focus of the scoping review, and included: (1) ‘migrant’ and specific migrant types (e.g. refugee, labor migrant); (2) Greater Mekong Subregion and the names of the five countries; (3) UHC, universal health coverage, health coverage, health financing, health insurance, out-of-pocket health expenditure, service delivery, and health equity. The references of retrieved publications were checked for relevant literature that was not otherwise identified by the search strategy. Current migration and UHC-related data in the GMS were compiled from relevant sources (e.g. World Bank, WHO). The status of ratification of key international legal instruments on international migration and health was examined for GMS member states, through reference to the United Nations Treaty Collection. Publications were reviewed for information relevant to the topic of migrants and UHC in the GMS, including the health of migrant populations in the subregion and challenges and opportunities for including migrants in UHC efforts. This paper develops the theme of health equity and migrants in the GMS and is informed by a report prepared in 2014 for the WHO Western Pacific Regional Office [19].

## Results

### Migration in the GMS

The GMS is experiencing increasing migration associated with rapid and uneven regional economic integration and economic growth, socio-economic vulnerabilities, and demographic disparities between countries [20,21]. The GMS countries have increased political and economic engagement through participation in the Association of Southeast Asian Nations (ASEAN) and the ASEAN Free Trade Agreement (AFTA) (with the exception of China), membership in the World Trade Organization (WTO), trade and foreign direct investment (FDI), and the creation of ‘economic corridors’ to facilitate cross-border trade [21,22]. Cambodia, Lao PDR, Myanmar, and Viet Nam are predominantly sending countries, while Thailand is predominantly a receiving country due to labour shortages and higher wage opportunities (see Table 1) [6].

**Table 1.** Immigrant and emigrant populations by country, GMS, 2015.

Country	Cambodia	Lao PDR	Myanmar	Viet Nam	Thailand
General trend	Sending	Sending	Sending	Sending	Receiving
Number of immigrants into country [10]	73,963	22,244	73,308	72,793	3,913,258
Females as % of immigrants [10]	46.1	46.3	45.2	42.1	49.7
Immigrants as % of national population [10]	0.5	0.3	0.1	0.1	5.8
Estimated number of refugees [10]	104	0	0	0	132,838
Estimated number of emigrants [11,12]	1,187,842	1,345,075	2,881,797	2,558,678	854,327
Main destination countries for emigrants	Malaysia, Thailand	Thailand	Thailand	Japan, Republic of Korea, Malaysia	Brunei Darussalam, Malaysia, Myanmar, Saudi Arabia, Singapore

Sources: [10–12].

An estimated 3–5 million labour migrants have migrated across borders in the Subregion, primarily for remunerated employment [21]. Irregular migrants in the GMS (i.e. people without required documents or permits) include large numbers of labour migrants who take on low-paid and low-status jobs and victims of human trafficking, especially women and children trafficked for forced labour and the sex industry [21,23,24]. Stateless persons, members of marginalised ethnic groups, and second-generation migrants without citizenship also resort to irregular migration [6,25]. Casual cross-border migration is widespread and typically occurs in remote areas [21]. Environmental and climate change-related displacement and migration are expected to increase in the Subregion, although much of this movement will occur within countries rather than across borders [20]. Thailand hosts approximately 133,000 refugees and asylum seekers, a large proportion of whom have fled Myanmar [10]. In addition, many displaced people live in border areas surrounding the refugee ‘camp’ areas in Thailand.

### International declarations and policy instruments

Migrants, as with all people, have a right to the highest attainable standard of health [26]. As enshrined in international human rights law, countries have an obligation to respect, protect, and fulfil the human rights of all individuals under their jurisdiction, regardless of their nationality of origin or immigration status [27]. For example, the 1990 International Convention on the Protection of the Rights of All Migrant Workers and Members of Their Families assures migrant workers access to ‘any medical care that is urgently required for the preservation of their life or the avoidance of irreparable harm to their health … on the basis of equality of treatment with nationals of the State concerned’ [28]. Yet despite such international declarations, stateless individuals often are denied ‘medical citizenship’ in their country of residence [25]. While GMS countries have ratified few international instruments relating to migration, all have ratified at least one international instrument protecting the right to health and other health-related human rights, thus providing a potential legal framework for action. There are increasing efforts to prioritise migrant rights in Asia, including a specific focus on migrants’ right to health (e.g. Joint United Nations Initiative on Migration, Health and HIV in Asia; 2007 ASEAN Declaration on the Protection and Promotion of the Rights of Migrant Workers; the 2011 Dhaka Declaration to promote migrant-inclusive health policies). The 10 ASEAN countries are now under pressure to make their health services more migrant-inclusive. The concept of ‘One ASEAN’ requires countries to facilitate the movement of people and trade and to move towards greater economic and social integration and harmonisation of legislation and standards [29,30].

### Health of migrants in the GMS

The conditions in which many migrants travel, live, and work carry significant risks for their health. Many migrants experience threats to their health due to inadequate access to social and health services, precarious migration status, lack of legal rights, restrictive immigration and employment policies, unsafe and exploitative working conditions, inadequate housing, low income, inadequate social security arrangements, social exclusion, separation from family, and anti‐migrant sentiments [7,31]. Health consequences include infectious diseases, occupational health hazards and injuries, poor mental health, non-communicable diseases (such as cardiovascular disease and diabetes), and maternal and child health problems. Infectious diseases including HIV/AIDS, tuberculosis, and malaria are major concerns, particularly among migrant workers entering Thailand and migrants in remote border regions [32–34]. For example, migrants in GMS border areas are at increased risk of malaria infection, as they are exposed to new vectors and malaria ecologies in host communities, and are typically under-served by health services [35]. Some migrants (e.g. victims of trafficking and low-skilled irregular migrants) are vulnerable to violence and exploitation, including sexual exploitation, which can lead to physical, mental, sexual, and behavioural health consequences including injury, depression, sexually transmissible infections (STIs), and harmful alcohol and substance use [25,36]. One report estimated that HIV rates were approximately two to three times higher among trafficked sex workers from Myanmar in Thailand (usually from Myanmar’s ethnic minority groups) than among Thai women working in the industry [37]. While there is inadequate understanding of migrant health vulnerabilities, it is clear that limited access to health services is a major challenge for migrants in the Subregion [29].

### UHC and migrants

As the United Nations Resolution ‘Transforming Our World’ highlights, ‘to promote physical and mental health and well-being and to extend life expectancy for all, we must achieve universal health coverage and access to quality health care’ [38]. Countries in the Asian region are at very different stages of UHC development, however, and many low- and middle-income countries are struggling to extend health coverage and to measure progress [39–41]. Accelerating progress on ‘coverage’ or ‘access’ are highly challenging goals, especially in low-income countries where supply and quality of services are inadequate, and ‘universal’ government health services usually require co-payments [19]. National health plans refer to UHC for ‘citizens’ or ‘the population’, the latter in practice usually excluding migrants (regular or irregular). There is a need to reframe debates in and beyond the health sector, to ensure that the commitment to ‘leave no-one behind’ – a feature of the SDG agenda – can be put into practice, including for migrants.

The discourse has moved beyond regarding UHC as mainly a matter of improving financial access and proposed indicators of progress now include measures of quantity, quality, and equity of services [42,43]. For example, the WHO Western Pacific Regional Action Framework views UHC as a whole-of-system approach to improving health system performance and health outcomes and lists five attributes of effective health systems: quality, efficiency, equity, accountability, and resilience [8]. It urges prevention of discrimination, including on the basis of migration status. The most basic measure of UHC progress however, remains problematic. Many of the world’s poor, particularly irregular migrants, are ‘invisible’ and not counted in official statistics [44]; for example, migrant populations are seldom mentioned in health impact assessments [10,45].

### GMS countries, UHC progress, and cross-border migrants

The capacity of GMS health systems to respond to migrant populations varies greatly. Table 2 summarises the migrant-inclusive features of health coverage in five GMS countries. Thailand has put the most effort into migrant health coverage; it has signed Memoranda of Understanding (MoU) with Cambodia, Myanmar, and Lao PDR to support labour migration management, which provide a foundation to support migrant access to health services in Thailand [46]. Viet Nam has developed health protection for citizens who emigrate for work. However, in the GMS overall (except Thailand), UHC remains weak for citizens let alone migrants. Migrant workers, and irregular migrants in particular, are not adequately covered and incur high out-of-pocket (OOP) payments: these populations are at-risk and disadvantaged in terms of health and have great need to be included in UHC. This is certainly advantageous from a public health perspective, but is politically and socially sensitive [29].

**Table 2.** Migrant-inclusive features of UHC in five GMS countries.

Indicators	Cambodia	Lao PDR	Myanmar	Viet Nam	Thailand
Policy and national-level frameworks	Constitution 2008 (Article 72) ‘All Cambodians’; Ministry of Health goal of UHC	National Health Strategy on UHC 2015–2020	National Health Plan UHC goal for citizens and designated ethnic groups	Health policies refer to citizens. *Health Insurance Law 2008* aims for UHC	*National Health Act 2002* set up UHC for those not covered by SHI schemes. National Health System Charter (Article 16) extends to everyone living in Thailand regardless of nationality.
Service models and coverage	HEFs cover 90% target of population (i.e. poor population) and 20% of national population	Limited SHI schemes. HEFs cover 41% of population	No specific programs	Govt. SHI covers 60% of population. Govt. subsidises premiums in poor areas	Mainly tax-financed: pay-roll tax SHI schemes, tax-based UHC for informal sector and poor. UHC covers 75% of Thai population who must register with district provider
UHC developments	HEFs scaling up across districts	HEFs being extended	UHC an accepted concept	Private health insurance allowed from 2011	Less OOP payment and increased out-patient visits for UHC beneficiaries
Migrant-inclusive features	District HEFs unlikely to enrol migrants. Some programs for emigrant workers, and some infectious disease programs	HEFs unlikely to enrol migrants. Some donor-funded programs for migrant workers	Not a national priority	Emigrant worker programs; joint government and donor infectious disease programs in border areas	MHI Scheme: legal migrant workers registered; irregular migrants can opt in. Targeted policies address migrant health: e.g. the National Master Plan for HIV/AIDS Prevention; Care and Support for Migrants and Mobile Populations (2007–2011); 2003 Thai Migrant Health Program
Current challenges	Huge challenge to fund and rebuild health system. High OOP payment	High OOP payment and inadequate health services	Huge challenge to improve health services. High OOP payment	Govt. services under-resourced. User fees for public and private health services	Migrant workers pay annual fees for MHI. Many irregular migrants do not register for MHI. MHI benefits are not portable and are less comprehensive than for Thai nationals

Sources: [5,8,8,14–16,29].

Notes: MHI: Migrant Health Insurance; HEFs: Health Equity Funds; OOP: out-of-pocket payments; SHI: Social Health Insurance; UHC: Universal Health Coverage.

All GMS countries are seeking sustainable ways to fund their health systems. Most are moving towards social health insurance (SHI) models, with subsidies for the poor funded from general tax revenue or by international donors [19]. Thailand’s government spends comparatively more per capita on health (PPP int. $) than other GMS countries (see Table 3). People in all GMS countries, however, encounter substantial OOP payments for health services and medicines (see Table 3).

**Table 3.** Socioeconomic, health system, and UHC indicators by country, 2012 or latest year.

Indicators	Cambodia	Lao PDR	Myanmar	Viet Nam	Thailand
Population (000s)	14,865	6,646	52,797	90,796	66,785
GNI per capita (Atlas method), current USD	1,360	1,650	1,270	1,890	5,370
Life expectancy at birth (years)	72	66	66	76	75
Under-5 mortality rate (per 1,000 live births)	40	72	52	23	13
Total health expenditure (THE) as % of GDP	5.6	2.8	1.8	6.8	4.1
Private expenditure on health as % of THE	77.4	50.6	84.1	54.8	22.3
OOP expenditure as % of private expenditure on health	80.3	78.2	93.7	83.2	55.8
Per capita total expenditure on health (PPP int. $)	129	75	23	227	372
Physicians (per 10,000 population)	1.7	1.8	6.1	11.9	3.9

GNI: gross national income.

Sources: [13–18].

### Access to health services by migrant type

#### Migrant workers

The flow of labour migration in Asia is from low-income to higher-income countries, but it is in the interests of both sides to support labour mobility and a flow of remittances [6]. Migrant workers have limited access to health services, however, and many workers are not aware of provisions even when these exist [47]. Thailand has made particular advances in enhancing migrant health coverage. In 2015, an estimated 3.9 million migrants were living and working in Thailand (see Table 1). Regular migrants – often labour migrants – are covered by the national Social Security Scheme (SSS). Further, the Migrant Health Insurance (MHI) scheme enrols regular migrant workers following pre-employment health screening, with annual premiums deducted from the migrant’s wage; health services are available only at the hospital where the migrant was registered, and some services available to Thai citizens are not accessible to migrants [29]. In 2014, approximately 1.6 million MHI cards had been issued. Thailand has also established specific policies/programs to address migrant health, including the Migrant Health Program. They focus on, but are not limited to, international labour migrants and seek to strengthen the migrant sensitivity of existing health services and develop migrant health services [48]. Other GMS countries with large overseas worker populations have legislation, bilateral MoU, and government structures to manage labour outflow including the welfare of migrant workers and their families. Viet Nam (predominantly migrant sending) has established a Law on Vietnamese Guest Workers (2007) which has provisions for mandatory vocational training, pre-departure language training and orientation, and the Overseas Jobs Support Fund [49].

#### Irregular migrants

Thailand has established policies and good practices that relate to health services for some irregular migrants. From August 2013, irregular migrants were allowed to register for the MHI with annual fees attached (1,500 Baht plus 500 Baht health check – or approximately 56 USD – as of 2014) [29]. However, irregular migrants face legal restrictions and limited access to health services, for example migrant workers in the fishing industry continue to be exploited and have little access to services and many irregular migrants (e.g. victims of trafficking) fear legal consequences from interacting with authorities and services [50,51]. Further barriers to the scheme’s uptake include the annual fees (for the health exam and insurance costs), lack of awareness of the scheme among irregular migrants, and reluctance of some hospitals to promote and implement the policy [29]. This illustrates the difficulty of achieving UHC even in a country with developed migrant health policies and programs. There is a paucity of research focused on access to health services among irregular migrants in other GMS countries, including because there are apparently few policies/programs that enable irregular migrants to access health services or that seek to ensure their health is protected while working overseas and upon return.

#### Victims of trafficking

Reliable data on trafficking in the GMS are unavailable due to its illegal and often undetected nature, but the Subregion is known to have diverse forms of trafficking. For example, men and boys from Cambodia, Lao PDR, and Myanmar are trafficked to work on fishing boats in Thailand, Malaysia, and Indonesia; women and girls from Lao PDR and Myanmar are trafficked for sex-work and domestic work in Thailand [52]. Victims of trafficking experience substantial barriers to health services due to fear of discriminatory treatment, fear of being reported to officials, fear of arrest and deportation, and the belief or knowledge that they are not entitled to or could not afford health services [34]. In 2004, leaders of all six GMS nations signed the MoU on Cooperation Against Trafficking in Persons in the GMS, an agreement that led to the Coordinated Mekong Ministerial Initiative Against Trafficking (COMMIT) [53]. It states a commitment to ‘providing all victims of trafficking with shelter, and appropriate physical, psycho-social, legal, educational and health-care assistance’. Yet it is primarily non-governmental organisations (NGOs) that work to meet the health and welfare needs of trafficked people in the Subregion [52]. For example, World Vision’s End Trafficking in Persons (ETIP) program in the GMS supports bilateral repatriation through case management, referral to services, and follow-up.

#### Refugees and asylum seekers

There are an estimated 452,000 refugees from Myanmar (the eighth-largest refugee source country, 2014–15), many of whom have fled to Bangladesh and Thailand [54]. Approximately 120,000 refugees and asylum seekers, as well as several thousand internally displaced people, live in nine so-called temporary camps along the Thai–Myanmar border [54]. Thailand is not a signatory to international conventions on refugees, and the Thai Government does not use the term ‘refugee’. Primary health services for refugees are funded by the Thai Ministry of Interior, Thai provincial/district authorities, NGOs, and international agencies [54]. During 2015, the election campaign in Myanmar raised the possibility for voluntary return of refugees and internally displaced persons; due to the landslide victory for the National League for Democracy (NLD), however, the potential for planned return of religious and ethnic minority refugees remains undefined.

#### Casual cross-border migrants

Casual cross-border migration in the GMS is widespread, i.e. movement of border residents between countries, either with or without passing border control checkpoints. Border regions are under-served with respect to health services. Migrants within border regions are likely to seek health services from unregulated private vendors, which can increase exposure to substandard drugs [55,56]. Artemisinin-resistant malaria has emerged in GMS border areas including the Cambodia–Thai and Myanmar–Thai border areas where there are high rates of migration [55]. Migration from and to areas where the disease is prevalent contributes to increased malaria transmission and incidence. A 2011 outbreak assessment in Attapeu Province in Lao PDR found that migrant workers accounted for 70% of confirmed malaria cases [57]. Cross-border collaboration and coordination are critical to vector control and improved malaria prevention, testing, and treatment for migrants [56]. The WHO Emergency Response to Artemesinin Resistance (ERAR) in the GMS targets high-risk migrant and mobile populations, including casual cross-border migrants. Containment strategies include scaling-up of prevention, diagnosis, and control activities that cover difficult-to-reach and mobile populations (e.g. screening and treatment at bus stations and work-site interventions), training community or village volunteers for diagnostic testing, referral and surveillance, treatment, coordinated cross-border activities, and improving drug quality and drug regulation [56].

## Discussion

### Improving migrant access to health services in the context of UHC

Health coverage for migrants in the GMS poses challenges for governments. Governments are seeking to improve the supply and quality of mainstream health services (e.g. through continued investment of funds, resources, and staff) and implement programs that address the health needs and health service access of migrants [7] and other vulnerable groups. National health laws and legal frameworks are required in order to extend social protection and health coverage, including for migrants [58].

Partnerships, networks, and multi-country frameworks are essential in order to ensure cross-border cooperation and collaboration on migrant health. Regional and subregional initiatives that address migrant health include: the Mekong Basin Disease Surveillance network; Joint United Nations Initiative on Mobility and HIV/AIDS (JUNIMA); WHO Mekong Malaria Program; WHO Regional Strategy to Stop Tuberculosis in the Western Pacific; bilateral collaboration and MoUs between Thailand and neighbouring GMS countries that focus on migrant workers; and the WHO Regional Action Framework on Universal Health Coverage. These examples of bilateral and regional cooperation highlight the growing focus on migrant health and UHC. Attention to cross-border migrant health is likely to increase, given the linkages to the SDGs and other international, regional, and national agendas. The ongoing move by countries towards integration under ASEAN, for example, will add momentum to extending health equity to their migrant populations [29].

While health service entitlements need to be expanded, improving the capacity of health services to address migrant health is equally important. Current access barriers for migrants include legal/administrative restrictions, language barriers, cultural constructs around illness and treatment, discriminatory attitudes amongst health service staff, and limited experience among health workers of migrant health issues. Increasing the migrant sensitivity of health services can support equivalence of care between migrant populations and local populations, e.g. through addressing language and cultural barriers, increasing the cultural competence of the workforce (both clinical and public health), and improving migrant health literacy. Universal access is a concept that goes beyond UHC, in that it requires health systems to remove geographical, financial, organisational, and socio-cultural barriers to care [59]. GMS governments face particular challenges in extending services to border areas, which are remote, mountainous, and often inaccessible. In Thailand, the Border Health Development Master Plan (2012–16) aims to improve the health and health services among people living in border areas (e.g. Thais, ethnic minorities, registered and non-registered migrants, displaced persons, and asylum seekers) [48]. It is important to further increase the quality and availability of health services in border areas where migrant and mobile populations reside.

Whether migrant health services should be separate or integrated into mainstream services is much debated. It is argued that health services that target migrant groups are important, particularly where infectious diseases (e.g. malaria, tuberculosis) pose substantial risks to migrants, the surrounding population, and beyond [60,61]. Currently, a few health initiatives target high-risk migrant and mobile populations in the Subregion, such as the WHO Emergency Response to Artemesinin Resistance in the GMS. Whether migrant health services/responses are integrated or separate, the involvement of migrant communities in decision-making (e.g. defining health concerns and service delivery needs) can reduce barriers to access, enhance integration, and improve population health. There is growing emphasis on creating opportunities for vulnerable groups, including migrants, to have a voice in health policy and practice [8]. Yet it is important to acknowledge that the health service users who are most reluctant and/or constrained in their engagement with services are usually the most disadvantaged [62].

Health coverage for irregular migrants is of particular concern, as they are rarely included in UHC frameworks yet comprise some of the most vulnerable migrant groups [29]. In order to achieve truly universal health coverage, entitlement and access to essential health services are necessary regardless of migration status. Governments will need to set the vision and direction to address inequities in health service access and to ensure the highest attainable standard of health among irregular migrants. This, however, remains a socially and politically sensitive issue.

There is limited research that examines migrant health in the GMS (other than Thailand). Further research at the country and subregional levels can contribute to understanding and supporting migrant inclusion in UHC efforts. Indicators to measure UHC progress should be disaggregated by relevant socioeconomic ‘stratifiers’, including migration status [4,63]. Migrant health and service use data can support governments to assess health disparities, make evidence-based decisions, and improve equity of service provision. Table 4 proposes solutions to overcome barriers to health coverage and health service access among cross-border migrants in the GMS.

**Table 4.** Cross-border migrants: examples of health system access barriers, problems, and solutions.

Type of barrier	Access problems	Access solutions
Human rights and laws	A country may not be a signatory to international and regional legal instruments on migration and health Migrants are not mentioned in the national constitution, national laws, or health policies	Advocate signing international and regional legal instruments on migration and health Make explicit reference to migrants’ health in health policies and plans, including diverse groups of migrants
Geographic	Migrant populations based in geographically remote areas Inadequate supply/standard of equipment and medicines Inadequate supply of staff in remote services	Send outreach services and staff to remote health facilities Improve supply/standard of equipment and medicine Cross-border programs in border regions that address joint health policy, financing, and health service delivery
Service delivery (availability, quality)	Restrictive eligibility for health services Lack of expert/relevant health knowledge on migrant health Limited data monitoring migrant health needs, health status, and service use	Lift or reduce restrictions for migrants; promote primary care services as entry point for migrant populations Train appropriate staff to understand and address migrant-related health issues, including both clinical and public health/health promotion workforce Introduce and improve migrant health data collection
Financial (affordability)	Migrants are not included in health insurance schemes Migrant health programmes are underfunded High user fees exist	Include coverage for migrants in health insurance schemes and HEFs Seek funding priority and new sources, including through private sector engagement, and regional and cross-border financing schemes Reduce OOP expenses and subsidise fees; introduce portal health insurance schemes for migrant workers
Sociocultural (acceptability, responsiveness)	Mono-cultural/mono-lingual services Lack of community engagement Health service/administrative staff have limited knowledge of social/cultural determinants of migrant health Migrants not literate and/or health literate Migrants have different cultural constructs around illness causation and treatment	Employ migrant/multicultural staff to act as intermediaries/facilitators Provide interpretation services Promote community participation Develop culturally tailored health programmes Provide cultural competency training and standards to staff Translate materials into formats that are acceptable to diverse migrant groups (including visual information and social marketing)

Sources: adapted from [19,60,61,64].

## Conclusions

The GMS will continue to experience migration in the coming years and decades. The emergence of a health equity agenda under international SDGs, and more focus on migrant health, provide an opportune time for GMS countries to develop migrant-inclusive health systems and strategies. It is of great importance that migrants are included. First, health coverage is not truly ‘universal’ unless migrants are included. Second, the health needs of migrants should be addressed as a matter of human rights. Third, it is in the GMS’s interests to protect the health of migrants as it pursues national and regional economic growth and social progress [29]. Yet for countries striving to achieve health equity, without specific efforts to include marginalised populations, there is a risk that poorer and disadvantaged segments of a population could be excluded from health service access and health promotion, leading to increasing inequities in health [59,63,65]. Health equity requires deliberate policy decisions that support health services and population health initiatives for irregular migrants and casual cross-border migrants, who remain amongst the most vulnerable and disadvantaged migrant groups. The ultimate goal of health equity is to improve population health outcomes. UHC represents an important opportunity to reduce barriers to health service access, extend coverage of curative services and health promotion efforts, and strengthen partnerships in order to improve the health of migrant populations.

## Paper context

Health coverage is only truly equitable and ‘ universal’ where migrants are included. This paper examines health needs and health care access among cross-border migrants in the Greater Mekong Subregion, and reviews associated policy responses. It argues that universal health coverage and health equity require deliberate policy decisions that support migrant access to health services, extend coverage of curative services and health promotion initiatives, and strengthen partnerships in order to improve the health of migrant populations.
